# Role of Electroconvulsive Therapy, Ketamine Infusion, and Deep Repetitive Transcranial Magnetic Stimulation in Treatment-Resistant Bipolar Depression: A Case Report

**DOI:** 10.3390/medicina60060936

**Published:** 2024-06-03

**Authors:** Keming Gao

**Affiliations:** 1 Mood Disorders Program, Department of Psychiatry, University Hospitals Cleveland Medical Center, Cleveland, OH 44106, USA; keming.gao@uhhospitals.org; Tel.: +1-216-844-2400; Fax: +1-214-844-2877; 2 School of Medicine, Case Western Reserve University, Cleveland, OH 44106, USA

**Keywords:** treatment-resistant bipolar depression, electroconvulsive therapy, ketamine infusion, deep repetitive transcranial magnetic stimulation, response, remission

## Abstract

*Background and Objectives*: Options for treatment-resistant bipolar depression (TRBPD) are limited. Electroconvulsive therapy (ECT) has shown efficacy in TRBPD. However, the cognitive deficits and memory concerns associated with ECT are problematic for a significant number of patients. It remains unclear what the next step is for patients with TRBPD who fail ECT. *Materials and Methods:* In this case report, we present a patient with TRBPD who sequentially received 12 sessions of brief-pulse right unilateral ECT, 22 sessions of ketamine infusion at 0.5–0.75 mg/kg for 40 min, and 39 sessions of deep repetitive transcranial magnetic stimulation (dTMS). *Results:* The patient had some benefit from ECT, but declined continuation of ECT due to memory concerns. The patient tolerated ketamine infusion well but had limited benefit. However, the patient responded well to acute treatment with dTMS and maintained relative stability for more than 2 years. *Conclusions:* This case suggests that patients with TRBPD who fail ECT and/or ketamine infusion might benefit from dTMS.

## 1. Introduction

The prevalence of treatment-resistant bipolar depression (TRBPD) remains unclear, although it is very common in clinical practice. Unlike treatment-resistant major depressive disorder (TRMDD) [1,2,3], the definition of TRBPD varies [4,5,6,7], and none has been widely accepted in the field of bipolar disorder research. The CINP Guidelines on the definition and evidence-based interventions for treatment-resistant bipolar disorder proposed the definition of the treatment-resistance of acute bipolar depression as “no significant reduction in MADRS (Montgomery-Asberg Depression Rating Scale)/Hamilton Depression Rating Scale scores or significant increase in YMRS (Young Mania Rating Scale)/MRS (Mania Rating Scale) scores or YMRS/MRS scores exceed 5” during treatment for 10–12 weeks [4]. An international expert panel on bipolar disorder defined TRBPD as failure of two of the following: olanzapine 10–20 mg/day plus fluoxetine 20–60 mg/day, quetiapine 300–600 mg/day, lurasidone 40–140 mg/day, and lamotrigine 200–400 mg/day; or failure of one of these four treatments and one of the following: lamotrigine 200–400 mg/day, valproate 1000–2000 mg/day, or lithium ≥ 0.8 mEq/L [5]. The duration of each treatment should last at least 4 weeks. The panel further defined “Multi-Therapy-Resistant Bipolar Depression” for those who meet TRBPD and fail at least 8 weeks of treatment with bupropion, an SSRI (serotonin reuptake inhibitor) or an SNRI (serotonin-norepinephrine reuptake inhibitor), a full course of cognitive behavioral therapy (CBT), and a trial of 12 bilateral ECT sessions. 

The lack of a “universal” definition of TRBPD has hindered the development of treatment strategies for patients who fail commonly used pharmacological agents, psychotherapies, and electroconvulsive therapy (ECT). Studies of ketamine infusion in the treatment of TRBPD showed promise but had limited efficacy in real-world populations [8,9]. A small study showed that repetitive transcranial magnetic stimulation (rTMS) was effective in bipolar depression (BDP), especially in bipolar I depression [10]. However, it remains unclear how these approaches can be applied to routine clinical practice. In this case report, we present a patient who had multi-therapy-resistant bipolar depression per definition of an expert panel [5] and sequentially received ECT, ketamine infusion, and deep repetitive transcranial magnetic stimulation (dTMS). The findings from this case will help clinicians rethink the role of ECT, ketamine infusion, and rTMS in the treatment of TRBPD.

## 2. Case Presentation

### 2.1. Initial Evaluation

History of present illness: Ms. A was a 51-year-old female referred to the Mood Disorders Program by her outpatient psychiatrist to manage her bipolar depression (BPD). At the initial evaluation, Ms. A reported that she had felt depressed again for about a month or so. Before that, she had a manic episode and was hospitalized involuntarily for 6 days. During the hospitalization, she was prescribed lurasidone 20 mg/day monotherapy but developed akathisia shortly after she was discharged home. She was not taking any medication at the time of the initial evaluation. 

During the evaluation, she reported that she felt depressed daily, most of the time, with a severity of 7–9 out of 10 (ten being the worst). She also felt hopeless, helpless, and worthless, with suicidal ideation (SI), but denied having a plan or intent for suicide. She stated that she could not kill herself because of her children. She also described a lack of motivation and pleasure, as well as insomnia. Her energy was low, and her concentration was poor. She reported that she was unable to read, although she was an active reader when she was not depressed. She also noted memory problems and poor appetite, with no desire to eat and a loss of five pounds in a month. She felt very irritable and easily agitated and often yelled at her significant other (SO). She denied having homicidal ideation (HI), auditory or visual hallucinations (AVHs), or paranoia. No delusional thought content was elicited. 

Past Psychiatric History: With the exception of the aforementioned hospitalization, she did not have previous psychiatric hospitalizations. She denied having previous suicide attempts or violence toward others. She reported having the first episode of depression when she was 15 years old after she learned that her mother was diagnosed with cancer. Her first episode of mania/hypomania started when she was 25 years old. She had symptoms consisting of hypomanic/manic episodes, including persistently increased energy and persistently elevated mood (euphoria) along with racing thoughts, increased self-esteem and confidence, decreased need for sleep, increased talkativeness, increased productivity, impulsive behaviors (reckless driving and drinking alcohol too much), and significant negative consequences. Her SO reported that her prior “manic” episodes were relatively short and did not cause significant negative consequences. She was unable to calculate how many depressive and manic/hypomanic episodes she had over the years. She stated that she had depression all her life, since she was teenager, during the assessment for hospitalization. 

She reported that she was diagnosed with bipolar disorder (BD) by some but not all of the psychiatrists whom she saw over the years. She received treatment with amitriptyline, nortriptyline, fluoxetine, sertraline, escitalopram, lithium, and methylphenidate from her previous mental health providers. Prior to the hospitalization, she had seen her outpatient psychiatrist for 9 years and received psychotherapy and escitalopram 20 mg per day for years. At the time of the hospitalization, she was taking escitalopram 15 mg per day and in the process of tapering off due to manic symptoms. Per her outpatient psychiatrist, she was prescribed aripiprazole 5 mg per day with escitalopram twice in the early state of the most current episode of mania and a manic episode 6 months earlier, but she only took one dose and stopped. She reported that she did not want to take antipsychotics or valproic acid because she feared weight gain and other potential side effects.

She attributed her most recent manic episode to menopause, which started 9 months ago. However, as per her outpatient psychiatrist, other factors, including one of children leaving for college several months earlier might have exacerbated the severity of the mania. During inpatient interviews, she reported that she felt “awesome”, “too happy, energetic, hypomanic”, and “ruling the world” initially, but gradually became “quick tempered”, was quick to “speak her mind” and experienced “getting into trouble with others”. Finally, she had significant negative consequences because of her impulsivity and poor judgement. Her SO reported she demonstrated erratic behaviors in last several weeks prior to the hospitalization. She totaled a car when they were on vacation; she left the house without letting others know; she drank alcohol excessively and got into arguments with others that resulted in serious negative consequences. Her outpatient psychiatrist reported that her behaviors were erratic, grandiose, akin to “taking over the world”, and excessively narcissistic in the past several weeks prior to the hospitalization, but she did not have any insight into her changed behavior. Her outpatient psychiatrist also reported to the inpatient team that she had olfactory hallucinations when she was manic at her 20s. Based on her symptoms and behaviors over the past several months, especially in the past several weeks, prior to the hospitalization, she was diagnosed with acute mania.

During the hospitalization, she received lurasidone 20 mg daily for 5 days and lorazepam 1 mg for anxiety. Her escitalopram was tapered off before she was discharged. Her manic symptoms quickly resolved. She was discharged home with lurasidone 20 mg/day. She reported that 3 days after discharge, she developed akathisia and stopped lurasidone. She did not want to take a medication for akathisia or to try another medication for her bipolar disorder. Her quick resolution of manic symptoms might be due to discontinuation of escitalopram and the nature of the illness. During the initial assessment after she was admitted, she stated that she felt like she was “crashing into depression now”, suggesting that her manic symptoms reached their peak before the hospitalization. 

Past medical history: She had a bilateral mastectomy and unilateral oophorectomy for the prevention of breast cancer due to elevated genetic risk. She reported a history of hypothyroidism without medication at the time of assessment. She also reported a history of low back pain and left knee pain due to generative joint diseases, a history of gastro-esophagus reflux disease without esophagitis, and a history of hyperlipidemia. At the time of her last admission, she did not have any physical complaints and did not take any medication for any medical condition. Her physical examination was unremarkable. Her laboratories at admission, including urinalysis, complete blood count, comprehensive metabolic panel, and renal function panel, were within the normal limits. The urine drug screen was negative. Thyroid stimulating hormone (TSH) was slightly elevated at 4.11 milliunits per liter (mlU/L). Two month later, the TSH level was 3.40 mlU/L (normal range 0.44–3.98 mlU/L). 

Family and Social History: She reported that her mother had depression and one maternal uncle had schizophrenia. She denied having a family history of BD, substance use disorder, or suicide attempts. She reported that her mother verbally and physically abused her when she was young. She has a Master’s degree in business management. She was married to her SO for 23 years at the time assessment and had two children. She was a stay-at-home mother at the time of assessment and had some problems with her SO because of the consequences of her recent manic episode. She had no problem with her children and denied having legal problems.

Psychiatric Comorbidity: She endorsed symptoms consistent with generalized anxiety disorder (GAD). She reported symptoms and behaviors consistent with alcohol use disorder (AUD), mild and in early remission at the time of assessment. She reported smoking cigarettes (2.5 packs per day for 5 years) when she was young, and she quit more than 20 years ago. She reported that she was diagnosed with bulimia nervosa for about a year when she was in college. 

Mental Status Exam: She was awake, alert, cooperative, and pleasant. She was well groomed and looked younger than her age. She was relaxed, calm, and able to make good eye contract. Her gait and muscle tone were normal. Her speech was clear, with normal rate, volume, and tone. Her fund of knowledge was adequate. Her mood was depressed and anxious. Her affect was appropriate most of the time, with intermittent smiling. Her thought process was goal-directed. She denied having SI, HI, AVH, or paranoia during the interview. Her attention and concentration were fair. Her short- and long-term memory were fine. Her insight and judgement were good, as she wanted to receive treatment for her depression. 

Diagnosis and treatment plan: She was diagnosed with bipolar I depression, recurrent, high-moderate to low-severe, along with GAD, AUD in early remission, and nicotine dependence in sustained remission. Her QIDS-16-SR (the 16-Item Quick Inventory of Depressive Symptomatology-Self Report) score was 18 points, indicating severe depression.

At the end of the evaluation, treatment options, including lamotrigine, quetiapine, and cariprazine monotherapy, combinations of mood stabilizers and antidepressants, and electroconvulsive therapy (ECT), were explained to her. She declined antipsychotics because of potential weight gain and other side effects. She was not interested in ECT because she did not feel that she needed it at the time. She agreed to try lamotrigine monotherapy given the minimal risk for weight gain. Lamotrigine was started at 25 mg at bedtime for 2 weeks, then 50 mg/day for 2 weeks, and then 100 mg/day, with a target dose of 200 mg per day. She also agreed to try zolpidem CR 12.5 mg at bedtime for sleep, because regular zolpidem did not keep her asleep in the past. She agreed to a follow-up visit in 4–6 weeks. 

### 2.2. Follow-Up Treatments 

First follow-up visit: She had the first follow-up about 2 weeks after the initial evaluation, which was earlier than initially planned. Her main reason for an earlier follow-up visit was the medication for sleep. She reported that she slept better with zolpidem CR 12.5 mg at bedtime but that she was concerned about addiction and dependence due to her recent history of AUD. She took quetiapine immediate release (IR) 50 mg at bedtime for sleep before the visit and responded well. She experienced dry mouth from quetiapine, without any other side effect. 

She reported that she felt better and less depressed shortly after the last visit because she slept better but continued feeling depressed daily all the time, with a severity of 5 out of 10 (ten being the worst) at the time of the visit. She denied feeling hopeless, helpless, or worthless and denied having SI, HI, AVH, or paranoia. She reported that her energy and concentration were better and she was able to read for a short period, but she did not have much change in motivation and pleasure. She no longer had memory concerns. She reported that her appetite increased without change in her body weight. She also felt less irritable and got along with her SO better. She was on lamotrigine 50 mg per day and quetiapine-IR 50 mg at bedtime at the time of the visit.

Second follow-up visit: She was seen 2 months after the first follow-up visit with her SO. She reported that she felt more depressed and restless because she was ruminating on what she did when she was manic. She reported her depression was 7 out of 10 (ten being the worst) all the time in the several weeks prior to the visit but denied feeling hopeless, helpless, or worthless and denied having SI, HI, AVH, or paranoia. She reported that she still slept well with quetiapine-IR 300 mg per day. Quetiapine-IR was increased from 50 to 300 mg because her depression did not improve much with 50 mg per day. She still reported daily lack of motivation and pleasure. Her energy was satisfactory. Her concentration had worsened again, although she was still able to read a book for a short period. Her appetite had decreased again, with some weight loss. She reported that she did not feel irritable but had to pace around all the time at home. She still got along with her SO, who confirmed that her depression and anxiety had worsened since the last visit. 

She still experienced dry mouth from quetiapine, without other side effect. Between the first and second visit, clonazepam 0.5 mg twice a day was also added for increased anxiety, and lamotrigine was titrated up to 200 mg at bedtime for depression. At the end of the visit, options including ECT, ketamine infusion, and an intensive outpatient program (IOP) were discussed and offered. 

Third follow-up visit: The third follow-up visit occurred 1 month after the last visit. She reported that she started the IOP in the Mood Disorders Program shortly after the last visit. The IOP consisted of psychoeducation, group cognitive behavioral therapy, peer support, need-based daily medication management, and attendance up to 5 days a week, with a minimum requirement of 3 days a week for each patient. At the initial evaluation for IOP, her PHQ-9 (Patient Health Questionnaire-9) score was 17 points, indicating severe depression. After 4 weeks in IOP, she reported that she did not feel any better, even with medication adjustments, including the addition of levothyroxine 25 mcg per day and bupropion extended release (XL) 150 mg per day for depression. Her CGI-S (Clinical Global Impression-Severity) score was 5 points at the end of the 4-week IOP assessment, suggesting that her depression was still severe after 4 weeks of IOP treatments. 

At the visit, she reported that she felt depressed and anxious daily all the time, with a severity of 8 out of 10 (ten being the worst) in the past several weeks. She stated she was unable to do anything at home and just sat in a chair rocking back and forth. During the visit, she stated that she wanted to get out of the depression and pain but did not want to die. She reported feeling hopeless, helpless, and worthless again and fearing her future, but she denied experiencing SI, HI, or AVH. She also reported that she felt someone whom she angered when she was manic might hurt her but denied other paranoia. She stated that she felt safe at home and on the street. She still slept well with quetiapine-IR 300 mg but continued feeling lack of motivation and pleasure. Her energy was low again. Her concentration, cognition, and memory had worsened. She stated that she could not write or spell. Her appetite continued to decrease, which resulted in 10 pounds of weight loss in 2 months. She reported that she did not feel irritable and got along with her SO, and she was able to take care of herself most of the time, but neglected her hygiene and appearance some days. 

Her medications were clonazepam 0.5 mg twice a day for anxiety, lamotrigine 200 mg at night for depression, quetiapine-IR 300 mg per day for depression and sleep, levothyroxine 25 mcg once a day, and bupropion XL 150 mg per day for depression. At the end of the visit, she wanted to consider ECT, but her SO believed that she did not have a fair trial of her medications. She and her SO agreed that she should stay on the same medications and continue IOP for a week or so before considering ECT. 

### 2.3. ECT and Post-ECT Treatments 

ECT treatment: About 2 weeks after the last visit, she started an acute ECT series with 12 brief-pulse right unilateral ECT (BP RUL ECT) treatments provided with the Mecta Spectrum 5000Q (Mecta Corporation, Tualatin, OR, USA). With the exception of the first treatment, with the parameters of pulse width of 1 millisecond (ms), frequency of 40 Hertz (Hz), duration of 2 s, and amplitude of 0.8 amperes (amp), the other 11 treatments utilized a pulse width of 1 ms, frequency of 90 Hz, duration of 4 s, and amplitude of 0.8 amp. These are the maximal settings of the ECT machine. With the exception of the second treatment, stimuli for all sessions were delivered once. The stimuli in the second session were delivered twice, with a setting of 1 ms, 60 Hz, 3 s, and 0.8 amp first, and a setting of 1 ms, frequency of 90 Hz, duration of 4 s, and amplitude of 0.8 amp about a minute later. With the exception of the third treatment (no motor seizure), the motor seizure durations of the rest treatments ranged from 21 s to 33 s. The EEG seizure was 13 s during the third treatment, and the rest ranged from 26 to 77 s.

The anesthetic for ECT was methohexital 60 mg (1.28 mg/kg) for all treatments. The muscle relaxant was succinylcholine 40 mg (0.85 mg/kg) for the first session and 20 mg (0.43 mg/kg) for the remaining treatments. Caffeine 480 mg i.v. (intravenous) injection and flumazenil 0.4 mg i.v. injection prior to stimuli delivery were added from the third treatment onwards to prolong seizure. Hyperventilation for 2 min prior to stimuli delivery was added from the fourth treatment onwards to prolong seizure. The hyperventilation was not used before adding caffeine and flumazenil because the ECT was performed during the peak of the COVID pandemic, and hyperventilation was not encouraged as per hospital guidelines. Ketamine 20 mg i.v. injection was added from the fifth treatment onward to prolong seizure and improve ECT efficacy. 

**Figure 1:**
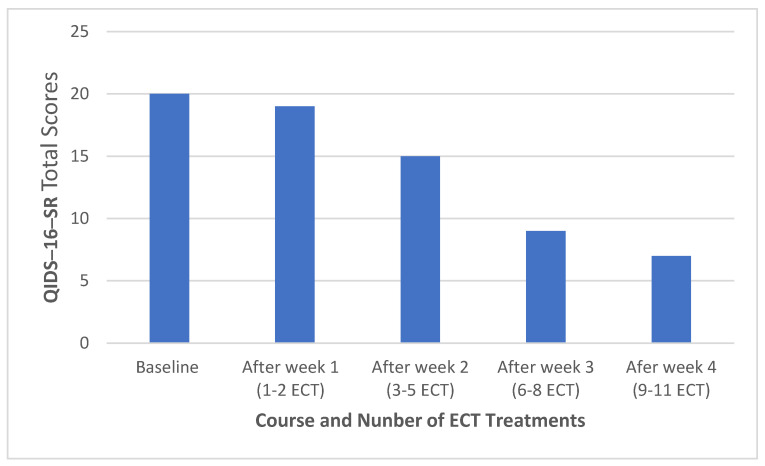
Changes in QIDS-16-SR total score during acute treatment with ECT. Abbreviations: QIDS-16-SR, the 16 item Quick Inventory of Depressive Symptomatology Self-Report; ECT, brief-pulse right unilateral electroconvulsive therapy.

Her progress during the ECT treatment is summarized in Table 1. The changes in QIDS-16-SR total scores are illustrated in Figure 1. Her medications before the first ECT treatment were clonazepam 0.5 mg twice a day for anxiety, lamotrigine 200 mg at night for depression, quetiapine-IR 300 mg per day for depression and sleep, and levothyroxine 25 mcg once a day and bupropion XL 300 mg per day for depression. She decided not to continue ECT after 12 treatments because she had some memory concerns during week 3, although her MoCA (Montreal Cognitive Assessment) score returned to baseline before her ninth treatment in week 4 (Table 1).

Medication management during the course of ECT: During the course of ECT, quetiapine-IR 50 mg was added for sleep as needed, and olanzapine 5 mg was added for decreased appetite and significant weight loss. Lamotrigine and clonazepam were held initially on the days before the ECT treatment. Lamotrigine was discontinued after the third treatment due to the short duration of motor and EEG seizure. Ongoing medications, including quetiapine IR 300 mg/day, levothyroxine 25 mcg/day, and bupropion XL 150 mg/day, were kept at the same doses as those prior to the first ECT during the entire course of ECT treatment. 

Post-ECT visit: Four weeks after stopping ECT, she had an office follow-up visit. During the visit, she reported that she had felt better with ECT and relatively stable for about 2 weeks, but she had felt very depressed again for about 2 weeks. She reported that she felt depressed daily about 75% of the time, with the severity of 7 out of 10 (ten being the worst), with feelings of hopelessness, helplessness, and worthlessness. She also reported that she felt scared because she did not want to go back to the depressive state prior to the ECT. She denied having SI, HI, or AVH and felt safe at home. She reported that she started rocking back and forth in a chair to soothe herself again, but this only lasted for less than an hour per day. She reported that she still enjoyed some activities and felt more pleasure than before the ECT treatment. Her energy was still good, but her concentration was still poor. Her memory was poor, and she believed it might be due to ECT. Her appetite was also not good, but she started gaining weight. She got along with her SO and was able to take care of herself most of the time, without neglecting her hygiene or appearance. 

Her medications were quetiapine-IR 300 mg/day, bupropion XL 300 mg/day, and levothyroxine 75 mcg per day for depression, clonazepam 1–2 mg per day for anxiety, and olanzapine 5 mg/day for decreased appetite and weight loss. The plan included continuing these medications, restarting lamotrigine, and considering ketamine infusion if depression continued worsening. 

### 2.4. Ketamine Infusion 

**Figure 2:**
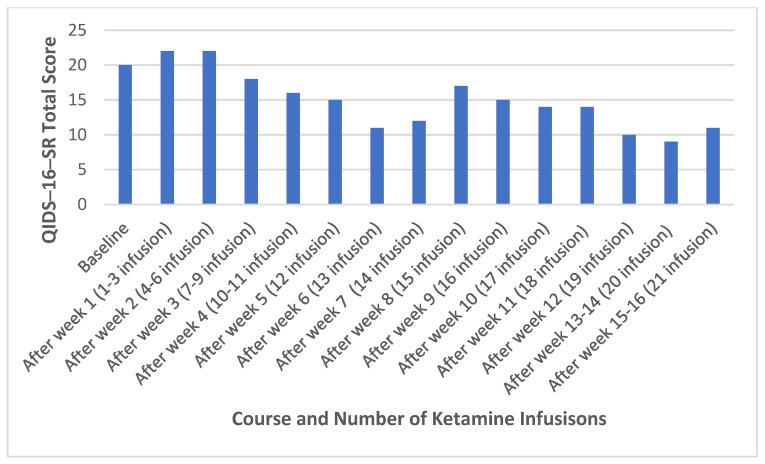
Changes in QIDS-16-SR total score during acute and continuation treatment with ketamine infusion. Abbreviations: QIDS-16-SR, the 16 item Quick Inventory of Depressive Symptomatology Self-Report.

About 2 months after stopping ECT, she received the first ketamine infusion at 0.5 mg/kg over 40 min. The dose of ketamine infusion was increased to 0.75 mg/kg from the fourth treatment onwards. She received 22 infusions, first three times a week for 3 weeks, then twice a week for 2 weeks, then weekly for 5 weeks, biweekly twice, and weekly again for the last treatment (Table 2). The changes in QIDS-16-SR total scores during the acute and continuation treatment are presented in Figure 2.

At the first ketamine infusion, she reported feeling very depressed daily, with 10 out of 10 severity (ten being the worst). She also reported feeling hopeless, helpless, and worthless and having a lack of motivation and pleasure, but she denied having SI, HI, AVH, or paranoia. She reported that she started having difficulty with sleep despite quetiapine 350 mg/day and clonazepam 2 mg per day. She continued to have no appetite and to lose weight even with olanzapine 5–10 mg per day. She reported increased rumination on what she did when she was manic.

Her medications during the course of ketamine infusion were quetiapine-IR 350 mg/day for depression and insomnia, clonazepam 1 mg twice a day for anxiety, bupropion XL 300 mg/day, and levothyroxine 75 mcg per day for depression. Olanzapine was increased from 5 mg to 10 mg because she continued to have no appetite and to lose weight. Fluoxetine 20 mg was added for her depression and anxiety and later was increased to 40 mg per day. The olanzapine and fluoxetine combination was the first medication approved for BPD and was effective in reducing depressive and anxiety symptoms [11]. Armodafinil 150 mg in the morning was added in week 12 of ketamine infusion because she continued feeling depressed and anhedonic. Armodafinil has shown some efficacy in the treatment of TRBPD [12]. However, she reported that she did not feel much better after 5 days of armodafinil 150 mg at the time of assessment before the 20th infusion. 

Her progress with ketamine infusion is summarized in Table 2. Overall, she tolerated ketamine treatment well. She reported feeling drowsy and floating on the bed during all sessions of the infusion and feeling euphoric, with a feeling of being “on top of the world” and/or thinking “I like this drug” in four infusions. She did not want to continue ketamine infusion for maintenance because she still had mild to low-moderate depressive symptoms during the course of ketamine infusion, as well as for financial reasons. Ketamine infusion was a self-pay service at the time of her treatments. Before she stopped ketamine infusion, the option of deep repetitive transcranial magnetic stimulation (dTMS) was discussed with her and her SO, although TMS was not approved for the treatment of BPD and was not covered by insurance companies in the United States. 

### 2.5. dTMS Treatment

About 1 week after stopping ketamine infusion, she received her first dTMS (Brainsway, Jerusalem, Israel) treatment. Her QIDS-16-SR total score was 17 points prior to the first treatment (Table 3). Before the treatment, she reported feeling alright overall but endorsed ongoing depressed mood, anhedonia, anxiety, and restlessness more than half of the time, as well as feeling hopeless, helpless, and worthless. She denied having SI, HI, AVH, or paranoia. She reported her energy was alright and concentration was fair most of the time. She stated she had to force herself to eat more to gain weight, even with olanzapine. She was able to take care of herself most of the time. She reported getting along with her SO, who was with her during the first treatment.

**Figure 3:**
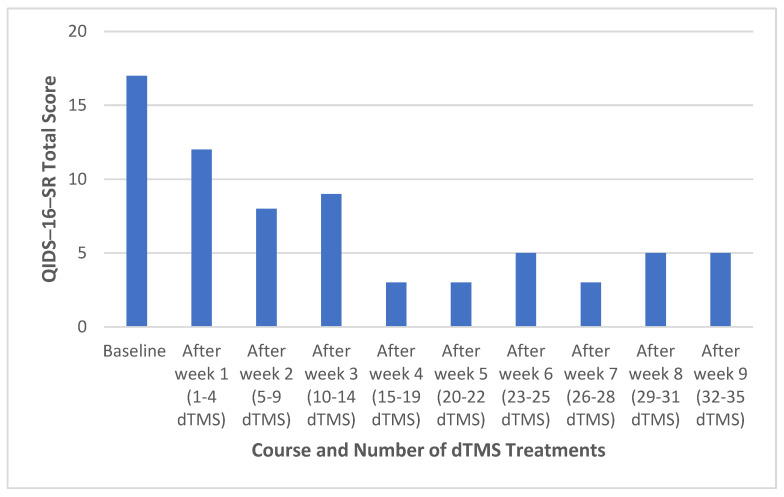
Changes in QIDS-16-SR total score during acute treatment with dTMS. Abbreviations: QIDS-16-SR, the 16 item Quick Inventory of Depressive Symptomatology Self-Report; dTMS, deep repetitive transcranial magnetic stimulation.

Her dTMS treatment started with 5 days a week for the first 4 weeks, followed by 3 days a week for the rest of the treatments authorized by her insurance company (Table 3). As per protocol for depression of the Brainsway, the coil to deliver the maximal magnetic output was placed over the left lateral prefrontal cortex (PFC). The motor threshold and the “best” location were mapped as per the manufacturer’s guidelines. The first treatment was administered at 100% of the motor threshold of the stimuli, with a frequency of 10 Hz, 2 s training stimuli, and a 20 s break. In the second session, the stimuli was increased to 110% of the motor threshold. From the third session onwards, the stimuli was 120% of the motor threshold (optimal intensity). Each treatment session lasted for 20 min. The changes in QIDS-16-SR total scores are presented in Figure 3. 

Her medications during the course of dTMS were quetiapine 350 mg/day for depression and sleep, and clonazepam 1 mg twice a day for anxiety; clonazepam was held the morning before dTMS. She also took bupropion XL 300 mg, levothyroxine 75 mcg, and armodafinil 150 mg in the morning for depression, and olanzapine 10 mg/day and fluoxetine 40 mg/day for depression, decreased appetite, and weight loss. She stopped dTMS treatment after 39 sessions because she had used up all the sessions authorized by her insurance company.

### 2.6. Post-dTMS Follow-Up Visits

First follow-up visit: About 5 weeks after the last dTMS, she reported that she felt very depressed again for a week. She stated that she felt good and stable for about 4 weeks before she felt depressed again. During the week prior to the visit, she had depressive mood and anhedonia and felt hopeless, helpless, and worthless, but she did feel excited and have good energy on the day of the visit. She denied having SI, HI, AVH, or paranoia. Her concentration was not good, but there were no changes in appetite or weight. She reported getting along with her SO, but they had some disagreements about her treatment. She reported that she was able to take care of herself most of the time and stopped olanzapine to avoid weight gain. She also wanted to taper off clonazepam because she worried about addiction and dependence. At the end of the visit, we discussed the options for treating the recurrence of depression, including resuming dTMS. 

Second follow-up visit: The second follow-up visit occurred about 2 months after the last visit. At the time of the visit, she reported that she had not had depression or anhedonia for about 2 weeks, although she felt anxious periodically. Her concentration was still not good, and her appetite was low, but she felt close to normal and had started daily exercise. She attributed her lack of depression and anhedonia in the last 2 weeks to her resuming dTMS at an outside location. She reported that her depression worsened after her last visit. She and her SO decided to have dTMS treatment (self-paid) at an outside location due to financial reasons. She reported that she had dTMS 5 days a week for 2 weeks and she felt much better at the end of the 2-week treatment. At the time of the visit, she was receiving dTMS twice a week. 

Her medications were quetiapine 350 mg/d for depression and sleep, clonazepam 0.5 mg twice a day for anxiety, and fluoxetine 40 mg, bupropion 300 mg, levothyroxine 75 mcg, and armodafinil 150 mg every morning for depression. She was unable to taper off clonazepam because of severe anxiety symptoms. 

Third follow-up visit: Four months after the last visit, she reported feeling pretty good and stable, with 2–3 dTMS sessions in a row on a monthly basis. She denied having depression or anhedonia and had felt normal for 2–3 months. Her medications were armodafinil 150 mg/d, quetiapine-IR 350 mg/day, clonazepam 0.5 mg twice a day, bupropion 300 mg/day, escitalopram 10 mg/day, and levothyroxine 88 mcg/day. After the last visit, her fluoxetine was switched back to escitalopram because she did not take olanzapine anymore, and she reported overall better efficacy with escitalopram than with fluoxetine. 

Fourth follow-up visit: Seven months after the last visit, she reported that she had felt good and relatively stable for about 6 months before she felt depressed again starting a month or so ago. She reported that she stopped dTMS about a month after the last visit. She felt relatively stable for about 6 months without maintenance dTMS. She reported that her main problem was anxiety instead of depression. She was worried about her children’s health and future as well as her own health. She had trouble falling and staying asleep, with somatic symptoms, including upset stomach. She started feeling more and more depressed because of her anxiety. 

At the time of visit, she reported feeling depressed daily most of the time, with moderate symptoms, and a little hopelessness and helplessness as well as anhedonia. She denied having SI, HI, AVH, or paranoia. Her energy was fair, but her concentration was not good. She reported her appetite had decreased, and she had lost 7 pounds in a month. She denied have psychomotor agitation, and she got along with her SO. She was able to take care of herself most of the time but neglected personal hygiene and exercise. She reported having to force herself to do the cooking and shopping. Her QIDS-16 score was eight points. She received a dTMS treatment at our clinic during the same office visit. She felt better after the second dTMS and stopped dTMS after the third treatment because she felt back to normal again. She did not have any change in her medications during the office visit and during the dTMS treatment.

Fifth follow-up visit: The fifth follow-up visit occurred 8 months after the last dTMS. She reported that her mood had been good and stable since the last dTMS treatment and did not have any symptoms of depression or anhedonia. Her medications were quetiapine-IR 350 mg/day, escitalopram 10 mg/day, bupropion XL 300 mg/day, levothyroxine 88 mg/day, and armodafinil 75–150 mg/day, as needed for anhedonia, decreased energy, and/or poor concentration. 

Sixth follow-up visit: Eight months after the last visit, she reported that she felt good and stable, until one and a half weeks ago. She reported feeling a little bit depressed and anxious again, which was triggered by her older child leaving for college and her youngest child being diagnosed with attention deficit hyperactivity disorder. She feared that her depression might worsen again, but she did feel better on the day of the visit. Her medications were the same as those at the last visit. 

As per communication with her SO, her depression did not worsen, and she felt back to normal shortly after the last visit. She remained in remission up to the time the case was finalized, which was about eight months after the last visit. 

## 3. Discussion

This case highlights the challenges in the treatment of TRBPD. Although the patient finally responded to dTMS and had remained stable for more than 2 years without maintenance of dTMS, it took the patient almost 2 years from the start of the depressive episode to reach remission and mood stability. The sequence of ECT, ketamine infusion, and dTMS in our case was based on the relative efficacy of each treatment modality relative to its control, insurance coverage, and the patient’s preference. It remains unknown if the patient would respond to dTMS if she received it before she tried ECT and ketamine infusion. 

Our case is unique because a single provider provided the services for medication management, ECT, ketamine infusion, and dTMS, and the treatment resistance level was sequentially determined. Lamotrigine was not approved by the US FDA (United States Food and Drug Administration) for the acute treatment of BPD, but it is recommended as a first-line medication for the acute treatment of BPD by the International Society for Bipolar Disorders (ISBD) and consensus from experts in bipolar disorders [5,13]. Quetiapine was approved by the US FDA for the acute treatment of BPD [14] and is recommended by ISBD as a first-line medication for the acute treatment of bipolar depression [13]. In addition, there is evidence supporting that the combination of quetiapine and lamotrigine is more effective than quetiapine alone for acute BPD [15]. Levothyroxine and bupropion are recommended as add-on therapies to those who fail mood stabilizer treatments [4,5]. Although the doses of levothyroxine 88 mcg/day and bupropion-XL 150 mg/day in our case were not “maximal” prior to ECT treatment, our case met the criteria of TRBPD regardless of the definition used [4,5]. With the failure of IOP, the level of treatment resistance of our case was likely higher than the commonly used criteria of TRBPD, such as failure of two or more medications from two different classes [4,5]. 

The remission rate of ECT in BPD is about 53% [16], but our case only achieved response (≥50% reduction in QIDS-16-SR score from baseline) after 11 ECT sessions. It was very unlikely, albeit possible, that the patient would achieve remission after the 12th ECT treatment. The progress of our case is consistent with a previous study of BPD with BP RUL ECT, in which a majority of patients achieved response (73.9%), but only a minority (34.8%) reached remission [17]. Bilateral ECT may increase the rate of remission [18], but our patient had memory concerns from BP RUL ECT, such that bilateral ECT was not an option. 

There is no study showing if patients who failed ECT would respond to ketamine infusion in TRBDP, but successful ketamine treatment after failure of ECT in TRMDD has been reported [19,20]. In our case, the patient only had a 20% reduction in depressive symptoms after the acute series of 2–3 times/week ketamine infusion at 0.5–0.75 mg/kg, but she did achieve response during the continuation phases (once a week to once biweekly) after 19 and 20 sessions (Table 2). Our results suggest that patients with TRBPD who respond to ECT treatment and cannot continue ECT due to memory concerns might have some benefit from ketamine infusion, but the onset of benefit from ketamine infusion might be slower than ECT (Table 1). Compared to patients with BPD who do not fail ECT and receive ketamine infusion, the onset of benefit from ketamine infusion in our case was much slower, and the magnitude of benefit was much smaller [9,21,22]. The limited efficacy in real-world patients [9] and the limited duration of efficacy [23] from ketamine infusion in TRBPD raise the question as to the role of ketamine infusion in the short-term and long-term treatment of TRBPD. 

The evidence of efficacy and safety of rTMS in TRBPD is lacking, although some studies suggest that rTMS is effective in reducing depressive symptoms compared to a sham [24]. However, the results were inconsistent [25]. In a routine clinical sample of patients with bipolar I or II depression, 44% of bipolar I patients and 28% of bipolar II patients achieved remission; 72% of bipolar I patients and 67% of bipolar II patients achieved response [10]. In a study of six patients with TRMDD who failed ECT treatment, two of six responded to dTMS [26]. In a small study of patients with bipolar I or II depression who failed ≥ 2 pharmacological interventions, the active dTMS was superior to the sham in reducing depressive symptoms, but the response and remission rates were not significantly different between the two arms [27]. Our case is the first report of a patient with sequentially determined TRBPD who failed ECT and ketamine infusion but fully responded to acute dTMS treatments, suggesting that some patients with BPD who fail ECT and/or ketamine infusion may still respond to rTMS, especially to dTMS. 

We could not determine how the early treatments with ECT and ketamine infusion affected the final response to dTMS. However, the course of response in our case to dTMS was similar to that of patients with TRMDD who were treated with dTMS and a sham after week two of treatment [28,29] and similar to active dTMS at week three. However, the reduction of depressive symptoms after week four of treatment in our case was much faster (Table 3) than the change in TRMDD [28,29]. Clearly, the placebo effect cannot be ruled out, especially in the second and third series of the dTMS treatments. 

## 4. Clinical Applications

ECT, rTMS/dTMS, and ketamine/esketamine are considered interventional treatments in psychiatry [30]. However, these three approaches differ in efficacy, risk, speed of onset, and durability. According to Berman and Ambrose [30], ECT has the best efficacy, rTMS has the best durability and lowest risk, and ketamine/esketamine have the fastest onset of action. Clearly, selecting the first treatment after failure of conventional antidepressants for TRMDD or mood stabilizers for TRBPD depends on the feasibility, affordability, logistics, and aim of acute treatment. What is the second step after failing the first one among the three interventional treatments? The same approach for selecting one over the other as selecting the first one may have to apply for the second treatment after the first one fails. Our case suggests that patients who fail the most efficacious intervention (ECT) may still respond to the least efficacious option (rTMS/dTMS) (Table 1 and Table 3). This finding is consistent with a retrospective study that found that a history of past ECT, regardless of responsiveness to ECT treatment, is not an independent factor for predicting TMS treatment outcome [31]. The effectiveness of TMS as a maintenance treatment after successful acute treatment with ECT supports the role of TMS in patients who respond to or fail ECT treatment in the course of acute and/or maintenance treatment of TRBPD or TRMDD [32,33]. 

In terms of ECT versus ketamine/esketamine, ketamine infusion at 0.5 mg/kg was as effective as ultra-brief pulse right unilateral ECT (UBP-RUL-ECT) in outpatients with TRMDD [34]. However, in hospitalized MDD patients who received 12 sessions of ketamine infusion at 0.5 mg/kg or 12 treatments of BP-RUL-ECT, ECT was significantly more effective than ketamine infusion in reducing depressive symptoms [35].

In a retrospective study of 24 patients with ECT-resistant TRMDD, six ketamine infusions at 0.5 mg/kg in 2 weeks yielded 33.3% with response and 20.8% with remission [36], suggesting that patients who fail ECT might benefit from ketamine infusion. Along with our case, this suggests that some patients with TRBPD who fail ECT due to lack of efficacy or adverse events might still benefit from ketamine infusion. 

For patients with TRBPD who fail ketamine infusion, brief-pulse right unilateral, bilateral, and bifrontal ECT may be a better option, although no data on ketamine infusion vs. ECT are available for BPD. Results from previous studies suggest that BP-RUL-ECT is more effective than UBP-RUL-ECT [37,38,39]. Therefore, the finding of ketamine infusion at 0.5 mg/kg as effective as UBP-RUL-ECT in TRMDD outpatients [34] should not be interpreted as an overall efficacy of ECT compared to ketamine infusion. In our case, we used BP-RUL-ECT, which was similar to the study of Estrand et al. 2022 [35]. The progress during the ECT and ketamine infusion in our case (Table 1 and Table 2) suggests that ketamine infusion may be less effective than BP-RUL-ECT in patients with TRBPD. In addition to ECT, TMS monotherapy or combination therapy with ketamine infusion may be an option for those who fail ketamine infusion alone, but large studies are needed to validate the results from case reports and small sample studies [40,41,42]. 

For patients who fail TMS, both ketamine infusion and ECT alone should be considered, although some studies found that patients who failed TMS responded to the combination of TMS and ketamine infusion [37,38,39]. Response to ketamine infusion after failure of TMS in TRMDD was reported [43]. Overall, ECT is more effective than TMS in TRMDD [40,41,42,44], but the efficacy of ECT depends on the electrode placement and parameters of stimuli. Previous studies have shown that bilateral or bifrontal ECT is more effective than BP RUL ECT [18,45,46], and BP-RUL-ECT is more effective than UBP-RUL ECT [38,39]. However, UBP-RUL-ECT has fewer side effects, especially related to memory and cognition, than other types of ECT. These differences should be taken into account when using ECT for TRBPD. 

## 5. Conclusions

In a sequentially determined TRBPD case, the ECT and ketamine infusion showed their limitations in the treatment of TRBPD. The unexpected efficacy and safety of dTMS in our case suggest that TMS may play a role in the treatment of TRBPD. However, the sequence of ECT, TMS, and ketamine infusion for maximal benefits as an option needs be determined with randomized adaptive-design multicenter studies. Our case also demonstrated challenges and highlighted the unmet need in the treatment of TRBPD.

## Figures and Tables

**Table 1:** Progress during the acute series with brief-pulse right unilateral electroconvulsive therapy.

Time of Treatment	Cumulative Number of Treatments	QIDS-16-SR Total Scores	Change from Baseline	MoCA Total Scores	Psychiatric Assessments
Baseline	n/a	20	n/a	24	Prior to the first ECT (electroconvulsive therapy) treatment, she reported feeling very depressed, a lack of motivation and pleasure, and anxious daily all the time, with a severity of 8 out of 10 (ten being the worst). Per herself, she did not do anything at home and just sat in a chair rocking back and forth. She also reported that she felt hopeless, helpless, and worthless and feared her future. She slept well with quetiapine 50 mg at bedtime. Her energy was low. Her concentration, cognition, and memory were not good, and she could not write or spell. She did not have an appetite, ate less, and lost 10 pounds in the last 2 months. She denied having suicidal ideation (SI), homicidal ideation (HI), auditory or visual hallucinations (AVHs), or paranoia.
During and after week 1	1–2 (Wednesday, Friday)	19	−5%	27	There was no change in mood and other depressive symptoms, although she reported feeling less anxious after the 1st ECT. She did not feel any difference after the 2nd ECT. She reported feeling very depressed, with suicidal ideation, but denied having a plan or intent.
During and after week 2	3–5 (Monday, Wednesday, Friday)	15	−25%	26	She did not feel much different after the second week of ECT treatments. She scored her depression as 7–8 out of 10 (ten being the worst). She reported feeling hopeless, helpless, and anxious but denied having SI, HI, AVH, or paranoia. She was not sure if ECT helped her or not. As per her significant other (SO), she seemed a little better.
During and after week 3	6–8 (Monday, Wednesday, Friday)	9	−55%	21	After the 6th treatment, she reported feeling better and less depressed and scored her depression 3 out of 10. She did not feel hopeless, helpless, or worthless anymore. After the week 3 treatment, she reported she had about 50% improvement and was able to force herself to eat more.
During and after week 4	9–11 (Monday, Wednesday, Friday)	7	−65%	24	During the week 4 treatment, she continued feeling better but started feeling forgetful. She reported feeling depressed intermittently, with a severity of 3 out of 10, and being active at home. As per her SO, she showed some “manic” symptoms, because she wanted to be on social media again. After the 11th treatment, as per her SO, she did break into her SO’s computer and was on social media for several hours over the weekend. No other manic symptoms were reported.
After week 5	12 Monday	n/a	n/a	n/a	Not available.

Abbreviation: MoCA, Montreal Cognitive Assessment; n/a, not available; QIDS-16-SR, the 16-item Quick Inventory of Depressive Symptomatology Self-Report. Note: A treating psychiatrist conducted psychiatric assessments prior to each treatment. The psychiatric assessments in the Table are a summary of these assessments. The patient filled out QIDS-16-SR prior to the first ECT treatment of the acute series (baseline) and then weekly prior to the first treatment each week. A trained member of the nursing staff administered MoCA prior to the first ECT treatment of the acute series and then weekly prior to the first treatment each week.

**Table 2:** Progress during acute and continuation treatment with ketamine infusion.

Time of Treatment	Cumulative Number of Treatments	QIDS-16-SR Total Scores	Change from Baseline	Psychiatric Assessments
Baseline	n/a	20	n/a	Prior to the 1st ketamine infusion, she reported feeling very depressed and anhedonia daily, with a severity of 10 out of 10 (ten being the worst). She reported ruminating on what she did when she was manic and feeling hopeless, helpless, and worthless on and off, but denied having SI (suicidal ideation). She also denied having HI (homicidal ideation), AVH (auditory or visual hallucination), or paranoia. She continued having problems with sleep, appetite, energy, and concentration, with weight loss even with olanzapine.
During and after week 1	1–3 (Monday, Wednesday, Friday)	22	+10%	She reported feeling a little better and did not stay in bed all the time after the first infusion, but she did not feel much different after the second one. She reported continuing to feel depressed most of the time, with moderate/severe symptoms and passive SI on and off, and she continued losing weight due to no appetite. She wanted to die because of psychological pain. Overall, she felt somewhat different, but not much, and even became worse over a long holiday weekend.
During and after week 2	4–6 (Monday, Wednesday, Friday)	15	−25%	She did not feel much different after the 4th infusion. She continued feeling very depressed with SI but denied having a plan or intent. She still isolated herself and stayed in bed most of the time. She forced herself to eat more. She did not want to be hospitalized and promised that she would change her behavior to prevent further deterioration and hospitalization. She reported feeling 20–30% improvement after the 5th infusion and believed ketamine infusion would work for her, although she continued feeling hopeless periodically, with moderate symptoms most of the time. Overall, she had some improvement after a total of 6 treatments.
During and after week 3	7–9 (Monday, Wednesday, Friday).	18	−10%	After the 7th infusion, she reported feeling markedly improved and scored her depression about 4 out of 10 (ten being the worst), but her QIDS-16 score was 16 points, indicating the depression was still severe. After the 8th treatment, she continued reporting feeling better, was able to stay out of her bedroom, had conversations with her husband, and started reading. She reported eating better and gaining some weight, but she did not do well over the weekend, isolated herself, and stayed in her room most of the time because of psychological factors and ruminating on her past.
During and after week 4	10–11 (Monday, Friday	16	−20%	She reported that ketamine infusion was helpful and made her strong enough to handle her depression and her guilt about her past. She reported sleeping well and eating more, with some weight again. As per her SO, her mood was unstable, and they were not sure if it was worth continuing ketamine infusion. She decided to have weekly ketamine infusion thereafter.
After week 5	12	15	−25%	She said she felt better and had 5 days of relatively “normal” and 2 days of severe depression. During those 2 days, she stayed in her bedroom most of time and rocked back and forth. During the relatively “normal” days, she was able to talk to her SO and her sister and stay out of her bedroom most of the time. She reported sleeping better, eating better, and gaining some weight. She denied having SI, HI, AVH, or paranoia.
After week 6	13	11	−45%	She reported reaching a plateau with ketamine infusion. Still felt depressed, anhedonic, and anxious intermittently, with mild to moderate symptom severity. She continued eating better, gaining weight, and sleeping well with medications.
After week 7	14	12	−40%	She reported feeling more depressed again but not as severe as before. She wanted to continue ketamine infusion because she felt it was helpful.
After week 8	15	17	−15%	She reported feeling alright and relatively stable, with daily moderate depression intermittently. She was able to engage in some family activities. Still ate better and slept satisfactorily with medications.
After week 9	16	15	−25%	She reported that her mood had “ups and downs” and generally improved with ketamine infusion.
After week 10	17	14	−30%	She reported feeling better and relatively stable but still felt depressed intermittently, with mild to moderate symptoms. She enjoyed some activities with her family. She continued eating well and gaining weight and slept well with medications.
After week 11	18	14	−30%	She reported feeling better and stable, with about 80% of her “normal”, but she still felt very depressed periodically.
After week 12	19	10	−50%	She reported feeling alright as well as eating well and sleeping well, and she believed ketamine infusion helped her to some extent. As per her SO, she did not do well in the past week.
After week 13–14	20	9	−55%	She reported feeling alright and did not feel much different compared to the previous week. As per her SO, ketamine infusion had reached the maximum benefit for her, and she was unstable, isolating herself at home, lacking motivation, and rocking back and forth in her room most of the time.
After week 15–16	21	11	−45%	She reported not doing well and had more bad days than good days. During bad days, she stayed in her room most of the time and felt more depressed and more anxious, but she denied having SI/HI/AVH or paranoia. She decided not to have more ketamine infusions.
After week 17	22	n/a	n/a	No assessment due to completion of treatment.

Abbreviation: n/a, not available; QIDS-16-SR, the 16 item Quick Inventory of Depressive Symptomatology Self-Report. Note: A treating psychiatrist conducted psychiatric assessments prior to each treatment. The psychiatric assessments in the Table are a summary of these assessments. The patient filled out QIDS-16-SR prior to each treatment. The first QIDS-16-SR score of the series (baseline), the first QIDS-16-SR score of each week during the acute series, and the QIDS-16-SR score prior to each continuation/maintenance treatment are presented in the Table.

**Table 3:** Progress during acute treatment with deep repetitive transcranial magnetic stimulation.

Time of Treatment	Cumulative Number of Treatments	QIDS-16-SR Total Scores	Change from Baseline	Psychiatric Assessments
Baseline	n/a	17	n/a	Prior to the first dTMS (deep transcranial magnetic stimulation), she reported continuing feeling depressed and anhedonic more than half of the time, feeling hopeless, helpless, and worthless intermittently, but denied having SI (suicidal ideation), HI (homicidal ideation), AVH (auditory or visual hallucination), or paranoia. As before, she stayed in her bedroom most of the time rocking back and forth in a chair. Her energy was low. Her concentration was poor. Her appetite was not good, even with olanzapine 10 mg/day. She also felt restless and easily irritable.
During and after week 1	1–4	12	−29%	She reported feeling alright and hopeful after the 1st dTMS because she did not have severe side effects. During the first week of TMS treatment, she felt optimistic, ate more, and slept better. Her energy and concentration were gradually better. She became more active at home and was able to participate in some family activities. She also realized she had been too critical to herself, which made her unstable. She reported having some anxiety throughout the week, but her irritability went away at the end of the week.
During and after week 2	5–9	8	−53%	During the second week of treatment, she continued reporting improvement in her mood, motivation, pleasures, interest, activities, energy, concentration, appetite, sleep, and anxiety. At the end of the week, she did not feel persistently depressed anymore, although she still had bad days. She also reported that her mood was more stable.
During and after week 3	10–14	9	−47%	Overall, she felt good and relatively stable. She believed that dTMS had been helpful, although she still felt depressed 15–20% of the time, with mild depression intermittently. She reported that she was able to soothe herself with reading, cooking, or social media. She felt close to “normal”. Her SO confirmed her reports.
During and after week 4	15–19	3	−82%	Prior to the first treatment of week 4, she reported feeling 100% better for more than a half of the time. During the entire week, she reported feeling close to 95–100% “normal” and had anxiety and depression occasionally.
During and after week 5	20–22	3	−82%	Prior to the first treatment of week 5, she reported not feeling well because she had a big argument with her SO over her use of social media, which caused her problems when she was manic. She admitted that she was addicted to social media and realized it was a problem for her. She reported feeling depressed and anxious for about 1 day, with a severity of depression of 7 out of 10 (ten being the worst), but was able to recover quickly and function well.
During and after week 6	23–25	5	−71%	Prior to the first treatment of week 6, she reported not feeling well because she was ruminating on what she did on social media last week, with the fear of ruining her reputation again. She felt miserable and discouraged but did not feel depressed. She reported that she “recovered” again after 2 days.
During and after week 7	26–28	3	−82%	During the entirety of week 7, she reported feeling very good and enjoying the time with her family, and she denied having other symptoms of depression. As per her SO, she was better and had more good days than bad days.
During and after week 8	29–31	5	−71%	During the entirety of week 8, she reported that she felt pretty good and stable and denied having depressive symptoms.
During and after week 9	32–35	5	−71%	During the entirety of week 9, she reported feeling close to “normal” and trying to think positive. She admitted that she tended to slide back to depression quickly, even there was no specific trigger.
During week 10	36–39	n/a	n/a	Prior to the first treatment of week 10, she reported she felt a little “dip” but was able to participate in family activities. During the week, she felt good and “normal”.

Abbreviation: n/a, not available; QIDS-16-SR, the 16 item Quick Inventory of Depressive Symptomatology Self-Report. Note: A treating psychiatrist conducted psychiatric assessments prior to each treatment. The psychiatric assessments in the Table are a summary of these assessments. The patient filled out QIDS-16-SR prior to the first dTMS treatment of the acute series (baseline) and then weekly prior to the first treatment each week.

## Data Availability

No data are unavailable due to privacy or ethical restrictions.
